# Fact or Fiction—Accelerometry Versus Self-Report in Adherence to Pediatric Concussion Protocols: Prospective Longitudinal Cohort Study

**DOI:** 10.2196/57325

**Published:** 2024-10-09

**Authors:** Carol DeMatteo, Sarah Randall, Josephine Jakubowski, Kathy Stazyk, Joyce Obeid, Michael Noseworthy, Michael Mazurek, Brian W Timmons, John Connolly, Lucia Giglia, Geoffrey Hall, Chia-Yu Lin, Samantha Perrotta

**Affiliations:** 1 Department of Rehabilitation Sciences, McMaster University, 1400 Main Street West, Hamilton, ON, L8S 1C7, Canada, 1 9055259140; 2 CanChild Centre for Childhood Disability Research, McMaster University, Hamilton, ON, Canada; 3 Department of Pediatrics, McMaster University, Hamilton, ON, Canada; 4 School of BioMedical Engineering, McMaster University, Hamilton, ON, Canada; 5 Department of Psychology and Behaviour, McMaster University, Hamilton, ON, Canada

**Keywords:** pediatric concussion, guidelines, adherence, return to school, return to sport, actigraphy

## Abstract

**Background:**

Concussion, or mild traumatic brain injury, is a growing public health concern, affecting approximately 1.2% of the population annually. Among children aged 1‐17 years, concussion had the highest weighted prevalence compared to other injury types, highlighting the importance of addressing this issue among the youth population.

**Objective:**

This study aimed to assess adherence to Return to Activity (RTA) protocols among youth with concussion and to determine if better adherence affected time to recovery and the rate of reinjury.

**Methods:**

Children and youth (N=139) aged 5‐18 years with concussion were recruited. Self-reported symptoms and protocol stage of recovery were monitored every 48 hours until symptom resolution was achieved. Daily accelerometry was assessed with the ActiGraph. Data were collected to evaluate adherence to the RTA protocol based on physical activity cutoff points corresponding to RTA stages. Participants were evaluated using a battery of physical, cognitive, and behavioral measures at recruitment, upon symptom resolution, and 3 months post symptom resolution.

**Results:**

For RTA stage 1, a total of 13% of participants were adherent based on accelerometry, whereas 11% and 34% of participants were adherent for stage 2 and 3, respectively. The median time to symptom resolution was 13 days for participants who were subjectively reported adherent to the RTA protocol and 20 days for those who were subjectively reported as nonadherent (*P*=.03). No significant agreement was found between self-report of adherence and objective actigraphy adherence to the RTA protocol as well as to other clinical outcomes, such as depression, quality of life, and balance. The rate of reinjury among the entire cohort was 2% (n=3).

**Conclusions:**

Overall, adherence to staged protocols post concussion was minimal when assessed with accelerometers, but adherence was higher by self-report. More physical activity restrictions, as specified in the RTA protocol, resulted in lower adherence. Although objective adherence was low, reinjury rate was lower than expected, suggesting a protective effect of being monitored and increased youth awareness of protocols. The results of this study support the move to less restrictive protocols and earlier resumption of daily activities that have since been implemented in more recent protocols.

## Introduction

Concussion, or mild traumatic brain injury, is a public health epidemic with an annual incidence of approximately 1.2% of the population [1]. According to the 2019 Canadian Health Survey on Children and Youth, head injuries or concussions had the highest weighted prevalence at 4.4% among children aged 1 to 17 years in Ontario, compared to other injury types [2].

In 2015, our research team developed evidence-based Return to Activity (RTA) and Return to School (RTS) protocols for children and youth with concussion [3,4]. These protocols (now updated) [5], and similar protocols based on the Sports Concussion Consensus statements [6,7], are the main management strategy for concussion recovery. It is important to determine whether youth adhere to these protocols before they can be evaluated in randomized control trials. At present, the most common method to assess adherence to the RTA/RTS protocols in youth is through self-report [6,8]. Literature suggests, however, that self-reported adherence estimates in youth are impacted by time since injury, age, mechanism of injury, receptivity to recommendations, and gender differences in activity [9-11]. To assess adherence, device-based measures of physical activity should be used, as they are reliable and minimize the bias associated with self-report [12-18]. As such, the primary aim of this study was to evaluate adherence to the RTA protocol using accelerometry and compare accelerometry-based adherence to self-report. The secondary objective was to evaluate postconcussion symptoms, recovery times, and rate of repeat head injury as well as to determine an association between adherence to RTA protocols and outcomes related to symptoms, repeat head injury, cognition, balance, quality of life, and depression. It was hypothesized that youth who were more adherent would have a lower incidence of repeat injury; shorter times to RTA; and better outcomes in quality of life, mental health, and cognition. Henceforth, youth will be used to refer to participants aged 5‐18 years in this study.

## Methods

### Ethical Considerations

This study was approved by the Hamilton Integrated Research Ethics Board (REB #14‐376). Informed assent/consent was obtained from participants and parents.

### Study Design

Participants were recruited from the local Hospital Emergency Department, community referrals from their primary physician, and rehabilitation or sports medicine clinics. Eligibility criteria included the following: a physician-diagnosed concussion within the past 12 months, being 5‐18 years of age, active symptomatology, and English-speaking ability. Youth were deemed ineligible if they had a confirmed brain injury requiring resuscitation, admission to the pediatric critical care unit, or surgical intervention, and if they refused to wear the ActiGraph. This prospective longitudinal cohort study had 3 measurement time points: recruitment/first visit; symptom resolution; and final visit, which occurred 3 months post symptom resolution or 6 months post enrollment if symptoms did not resolve within the study time frame. This investigation consisted of various outcomes, including electroencephalogram, [19,20] magnetic resonance imaging (MRI) [21], cognition [22,23], and sleep [24], which were published previously. Data on self-reported adherence, and adherence to RTS protocols specifically, were published by DeMatteo et al [8].

### Assessment of RTA and RTS Protocol Stages and Symptoms

The CanChild protocols [3,4] consist of 6 stages of RTA and 5 stages of RTS, made with reference to the Zurich guidelines [25]. Youth were advised that no high intensity physical activity or contact sports was permitted while they were symptomatic. They were also informed that “rest” does not equate to social isolation or sensory deprivation. Once recruited, youth received the ActiGraph and the 2015 CanChild protocols [3,4] immediately. Participants completed surveys every 48 hours using REDCap (Research Electronic Data Capture; version 14.5.10, 2024; Vanderbilt University)—a browser-based data management application [26]. The surveys included the Post-Concussion Symptom Scale (PCSS) [27], RTA/RTS stages, and an assessment of cognitive activity [3]. The PCSS [27] is common across concussion evaluations [7] and consists of a 22-symptom checklist scored on a 0‐6 Likert scale. This was adapted for younger children using a dichotomous yes/no scale [28]. The cognitive scale assessed cognitive activity on a scale of 1‐5 and was adapted from Brown et al [29]. The second in-person visit occurred at symptom resolution. The label of symptomatic or nonsymptomatic was based on the return of participants’ self-identified current reporting of symptoms to their preinjury symptom status.

### Measurement of Adherence to Protocols

To assess physical activity in the RTA protocols, youths were outfitted with an ActiGraph Gt3X waist-worn monitor accelerometer (ActiGraph LLc). The ActiGraph accelerometer provides a high-resolution measure of the duration, intensity, and frequency of movement and is validated for use in youth [12-17]. Participants were provided standardized instructions on how to wear the accelerometer and to record times of nonwear in a log diary. Accelerometry data were downloaded into 30-second epochs and visually inspected by trained personnel to ensure wear times matched those reported by participants. The data were cleaned to remove any nonwear periods or spurious data using ActiLife (version 6.13.4; ActiGraph LLc). The 30-second epoch was selected for analyses, as shorter epochs are more accurate to measure exercise intensity during intermittent physical activity [30]. Only valid days, defined as at least 6 hours and no more than 19 hours of wear time, were included in the analysis. Activity count data were then scored for analysis of adherence. To do this, daily time spent being sedentary or engaging in light physical activity (LPA), moderate activity, vigorous physial activity, and moderate-to-vigorous physical activity (MVPA) were calculated using the Evenson et al [14] cut points. Youth were considered adherent if there was 80% adherence to the physical activity requirements for the corresponding stage of the RTA protocol. Only participants who had complete actigraphy data were included in analyses (n=84). For stage 1, unlimited LPA was permitted, MVPA was limited to ≤2.5% of wear time and no consecutive bouts of ≥5 minutes at any intensity were recommended. In stage 2, baseline activity observed in stage 1 was permitted, as well as an extra 30 minutes of LPA, but no consecutive bouts ≥5 minutes of MVPA were recommended. In stage 3, baseline activity observed in stage 2 was permitted, as well as an extra 60 minutes of MVPA and upto two 15-minute bouts of MVPA. Only adherence to RTA stages 1‐3 were assessed with accelerometry because these stages had quantifiable activity amounts defined in the RTA protocol [4], and RTS did not have objective adherence data.

Subjective reported adherence to the RTA/RTS protocols was based on the following criterion: if participants received a label of “yes” to the questions asked by the research personnel: “Has the child been following the RTA/RTS guidelines correctly?” based on a self-reported progression through the RTA/RTS stages being associated with a decreasing self-report PCSS score. [8] Participants were categorized separately for RTA and RTS. Participants labeled as “Did not adhere” to both RTS and RTA, as well as participants labeled as “Adhered” to one but not both RTA and RTS protocols, were deemed “Did not adhere.”

### Standardized Neurocognitive, Depression, Quality of Life, Coordination, and Balance Tests

Participants completed the short form of the Children’s Depression Inventory (CDI) [31]; the KIDSCREEN-52 [32]; Immediate Post-Concussion and Cognitive Test (ImPACT) [33]; and subsets of balance, bilateral coordination, running speed, agility, and strength from the Bruininks-Oseretsky Test of Motor Proficiency Second Edition (BOT-2) [34] at each in-person visit.

### Statistical Analyses

Demographic and injury data are presented as mean (SD), recruitment details are reported in percentages, and the PCSS score is reported as median, which better reflects the data due to a few outliers. Adherence for each participant was calculated for stages 1‐3 of RTA as described above. Adherence was determined a priori to be considered the primary predictor of outcomes, but as the adherence rate was very low, alternative analyses were performed (as explained in the *Results* section). The ActiGraph calculated adherence for RTA was then compared to the self-reported rating of adherence for RTA, and agreement was assessed using Cohen κ.

The rate of repeat head injury was calculated as a percentage of total injuries. A Mann-Whitney *U* Test was performed to assess time to RTA stage 3 and 6 and time to symptom resolution for those who adhered or did not adhere [15,35]. Only participants who reported continued symptoms up until 3 months post symptom resolution, self-reported adherence to RTA/RTS, and reported the final stage of RTA/RTS were analyzed. Participants who responded “no” to the question “On more than one occasion, have you had any symptoms of concussion in the last two weeks?” were given a PCSS score of 0. Significance was set at *P*=.05. Scores for the BOT-2, CDI, ImPACT, and KIDSCREEN-52 were reported as mean (SD) and median. All the data were tested for normality using the Shapiro-Wilk test. Time to symptom resolution, time to stages, and PCSS scores were not normally distributed. Data were analyzed using SAS (version 9.4; SAS Institute) and SPSS (version 23.0; IBM Corp), with significance set at *P*<.05.

## Results

### Overview

Of the 139 participants who consented to the study, 107 (76.9%) participants completed follow-up assessments, 12 (8.6%) participants were lost to follow-up, and 20 (14.3%) participants withdrew from the study. Of the 20 participants who withdrew, 7 (35%) participants did so immediately after consent, 12 (60%) participants withdrew after the first in-person visit, and 1 (5%) participant withdrew before the final visit.

The cohort included 64 (46%) boys and 75 (54%) girls with a median age of 13.4 years. A total of 103 (74.1%) participants sustained their concussion via a sports-related injury, with most injuries obtained during recreational play (n=29, 28%) and ice hockey (n=26, 25%). This was the first concussion for 58.3% (n=81) of participants (Table 1). The median time from injury to the first visit was 7.8 days (mean 34.8 days, minimum 2.9 hours, and maximum 320.9 days). The mean time from injury to symptom resolution visit was 95.4 (SD 43.4) days for first in-person visits and 162.6 (SD 75.7) days for final visits.

Of the participants who remained in active enrollment (n=114), 16 (14%) participants did not achieve symptom resolution in the 6-month follow-up period (Table 1). Median time to symptom resolution was 16 days (Q1-Q3: 8-28; mean time 27, SD 33 days).

The rate of participants having another concussion during the follow-up period was 2% (n=3).

**Table 1.** Participant demographics, symptom resolution, and rate of reinjury (N=139).

Demographics		Values
**Age (N=139)**		
Mean (SD)		13 (2.85)
Median (Q1-Q3)		13.4 (10.9-15.2)
**Sex (N=139), n (%)**		
Male		64 (46)
Female		75 (54)
**Number of previous concussions (N=139), n (%)**		
0		81 (58.3)
1‐2		45 (32.3)
3‐5		8 (5.8)
>6		4 (2.9)
**Mechanism of injury (N=139), n (%)**		
Sports/recreational play		103 (74.1)
Non–sports-related injury/fall		22 (15.8)
Assault		5 (3.6)
Motor vehicle collision		4 (2.9)
Other		3 (2.1)
**Post-Concussion Symptom Scale baseline score (n=131), median (Q1-Q3)**		36 (17-56)
**Achieved symptom resolution (n=114), n (%)**		
Symptom-free within 7 days		2 (1.7)
Symptom-free in 8‐14 days		16 (14)
Symptom-free in 15‐28 days		31 (27.2)
Symptom-free in 29‐89 days		35 (30.7)
Symptom-free in >90 days		14 (12.3)
Never achieved symptom resolution		16 (14)
**Withdrew/lost to follow-up prior to symptom resolution (n=25), n (%)**		
Unknown		11 (44)
Past 30 days		3 (12)
Past 60 days		2 (8)
Past 90 days		9 (36)
**Rate of reinjury (N=139), n (%)**		3 (2.1)

### ActiGraph Adherence Evaluation

Based on the participant analysis, 13% (4/30) of participants adhered to stage 1; 11% (8/74) adhered to stage 2; and 34% (17/50) adhered to stage 3 (Table 2). Of note, only 1 participant from this cohort (N=139) adhered to all 3 stages.

**Table 2.** Days of participant adherence per stage to the Return to Activity (RTA) protocols based on actigraphy (N=139).

Participant ActiGraph adherence data (N=139)	Stage 1	Stage 2	Stage 3
**Wear time criteria met, n (%)**	30 (21.6)	74 (53.2)	50 (36.0)
Adhered	4 (13.3)	8 (10.8)	17 (34)
Did not adhere	26 (86.7)	66 (89.2)	33 (66.0)
**Wear time criteria not met or PCSS^a^ and stage data not available, n (%)**	109 (78.4)	65 (46.8)	89 (64.0)

a PCSS: Post-Concussion Symptom Scale.

ActiGraph data with sufficient wear time and the corresponding PCSS score and RTA stage were considered complete and then analyzed in 30-second epochs for 80% adherence to stages 1, 2, and 3. Participants were labeled as “Adhered” to each stage if they had at least 1 day in adherence to ActiGraph cut points for stage 1, 2, or 3. Participants were given a final label of “Did not adhere” if they did not meet the cut points corresponding to stage 1, 2, or 3.

### Subjective Reported Adherence

Of the 105 participants with self-reported data, 59 (56.1%) participants adhered to the RTA protocol [11], 56 (53.3%) adhered to the RTS protocol, and 50.4% (n=53) were adherent to both protocols (Table 3).

**Table 3.** Subjective adherence reported for the Return to School (RTS) and Return to Activity (RTA) protocols (n=105).

Adherence	RTS	RTA	RTS and RTA
Adhered, n (%)	56 (53.3)	59 (56.1)	53 (50.4)
Did not adhere, n (%)	49 (46.6)	46 (43.8)	52 (49.5)

### Objective ActiGraph Versus Subjective Self-Report

Cohen κ was performed to determine if there was agreement between actigraphy and self-reported adherence to the RTA protocol. There was no statistically significant agreement between the two measures (κ=0.49, 95% CI 0.32‐0.66*; P*=.57; Table 4). Among the 84 participants with both ActiGraph and self-reported data, there was 48% (n=40) agreement between the two. A total of 36 (43%) participants self-reported adherence to the RTA protocol but failed to meet the ActiGraph adherence cut points.

**Table 4.** Agreement of adherence between ActiGraph versus subjective report (n=84). There was no statistically significant agreement between the two measures (κ=0.49, 95% CI 0.32‐0.66*; P*=.57).

Subjective report	Actigraphy		
	Adhered, n (%)	Did not adhere, n (%)	Total, n (%)
Adhered	16 (19)	36 (43)	52 (62)
Did not adhere	8 (9)	24 (29)	32 (38)
Total	24 (29)	60 (71)	84 (100)

### Time to Symptom Resolution and RTA/RTS Completion

Those with subjective reported adherence to the RTA protocol had a significantly shorter time in days (median=13) to symptom resolution than those who did not subjectively adhere (median=20; *U*=724.50; *P*=.03; Table 5).

The difference in time to symptom resolution was assessed, using a Mann-Whitney *U* test, for participants who self-reported adherence and nonadherence to RTA and RTS protocols (n=90). Time to symptom resolution was calculated as the time from initial injury to symptom resolution. Only participants who had a date of symptom resolution verified by research personnel were included in the analyses.

There was no statistically significant difference in time from injury to RTA stage 3 (*P*=.61) or stage 6 (*P*=.24) for participants who self-reported adherence or nonadherence (Tables6  7).

**Table 5.** Time to symptom resolution and Post-Concussion Symptom Scale (PCSS) score for youth with concussion based on subjective adherence or nonadherence to Return to Activity (RTA) and Return to School (RTS) protocols (n=90).

Variable		RTA			RTS		
		Adhered	Did not adhere	*P* value	Adhered	Did not adhere	*P* value
**Time to symptom resolution (days)**							
Total, n	49	41	—^a^	47	43	—
	Mean (SD)	23.0 (30.7)	32.9 (36.6)	—	21.6 (26.1)	34.0 (39.7)	—
	Median	13.0	20.0	—	13.0	17.0	—
	Minimum	2.0	2.0	—	2.0	2.0	—
	Maximum	157.0	174.0	—	157.0	174.0	—
	Mean rank	40.15	51.89	.03^b^	41.38	50.0	.12
**PCSS score at symptom resolution**							
	Total, n	40	37	—	44	42	—
	Mean (SD)	2.4 (10.6)	7.4 (15.1)	—	4.5 (11.7)	7.4 (14.6)	—
	Median	0.0	0.0	—	0.0	0.0	—
	Mean rank	37.06	41.09	.29	42.90	44.13	.78

a Not applicable.

b Statistically significant.

**Table 6.** Time in days to return to activity (RTA) for youth with concussion based on subjective adherence or nonadherence to the RTA protocol (n=105).

Time to RTA (days)	RTA stage 3			RTA stage 6		
	Adhered (n=34)	Did not adhere (n=19)	*P* value	Adhered (n=41)	Did not adhere (n=36)	*P* value
Mean (SD)	56.4 (60.1)	38.8 (54.9)	.61	59.9 (41.5)	63.1 (65.3)	.24
Median	29.8	15.2	—^a^	47.3	31.6	—
Minimum	7	6	—	12	11	—
Maximum	247	221	—	156	276	—

a Not applicable.

**Table 7.** Time in days to return to school (RTS) for youth with concussion based on subjective adherence or nonadherence to the RTS protocol (n=105).

Time to RTS (days)	RTS stage 3			RTS stage 5		
	Adhered (n=36)	Did not adhere (n=22)	*P* value	Adhered (n=47)	Did not adhere (n=41)	*P* value
Mean (SD)	28.3 (39.9)	13.2 (8.7)	.06	65.2 (57.9)	58.9 (64.2)	.05
Median	13.8	10.7	—^a^	45.7	27.5	—
Minimum	5	5	—	11	7	—
Maximum	199	44	—	252	253	—

a Not applicable.

### PCSS Score at Symptom Resolution

The difference in the average number of symptoms as reported on the PCSS or postconcussion system inventory at stage 5 of RTS and stage 6 of RTA was assessed, using a Mann-Whitney *U* test, for participants who self-reported adherence and nonadherence to RTS/RTA protocols (n=86). There was no statistically significant difference in the PCSS score at stage 5 or stage 6 of RTS (*P*=.78) and RTA (*P*=.29), respectively, for participants who adhered or did not adhere to the protocols (Table 5).

### Adherence and Nonadherence: Depression, Quality of Life, Neurocognitive, and Balance Tests

The KIDSCREEN-52 physical and psychological well-being subsections scores improved from the first to final visit across most participants. The scores were considered “high,” demonstrating that participants felt they were physically fit and healthy and viewed life positively [29]. Participants’ CDI total T-score decreased for symptoms of depression from the first to the final visit (*P=*.33), where scores were in the average/low range (<60). Across all 3 visits, most participants scored in the “average” category. From the first to the final visit, the ImPACT subsection scores increased, suggesting an improvement in cognitive performance (*P=.*13).

There was no significant difference in the BOT-2, CDI, and KIDSCREEN-52 total or subsection scores between those who reported they subjectively adhered or those who did not adhere to the RTA and RTS protocols. There was a significant difference in the ImPACT Impulse Control Composite Score at the final visit for those who adhered to the RTA protocol (mean score 7.3, SD 5.1) versus those who did not adhere (mean 11.9, SD 11.7; *P*=.04).

## Discussion

This prospective cohort study examined adherence to the RTA protocol, the rate of reinjury and time to symptom resolution among youths with concussion. It is one of the few investigations that has assessed physical activity in youth with concussion using accelerometry [36]. Our findings indicate that youth have lower adherence to RTA stages, as measured by accelerometry, when physical activity is more restricted, with adherence improving as more activity is allowed. Actigraphy analysis showed that 13% of participants were adherent to the RTA stage 1; 11% were adherent to stage 2; and 34% were adherent to stage 3. Huber et al [37] examined collegiate and high school football players post concussion using the Fitbit Charge HR. The authors found that athletes with concussion had a great deal of variability in activity levels the first few days post injury, suggesting differences in how the athletes interpreted “rest.” Although in Huber et al [37] the activity monitors were worn for only 2 weeks, their findings are similar to ours in that there is lower adherence in the early stages. In our study, the generally low adherence rate was not conducive to any statistical prediction analyses or modeling, as the study had set an a priori standard of 80% compliance to qualify as “adherent” to predict whether these adherent youth would have better outcomes. This required examining the data in other ways, resulting in compelling findings. First, we observed that the PCSS score decreased as youth progressed through the RTS/RTA protocols [8] and remaind low at the final stage of RTS/RTA, despite low adherence according to activity monitoring. We also observed a rate of reinjury of merely 2%, which is lower than the rates presented in the literature [38,39]. In addition, the same referral-based sample of patients at the McMaster Acquired Brain Injury Concussion Clinic in the 2013‐2014 period (before the RTA/RTS protocols were first introduced) documented a reinjury rate of 37% among the 464 youths followed clinically. Notably, 36 (43%) participants self-reported adherence to the RTA protocol but failed to meet the actigraphy cut points. This suggests that they believed they were following the activity recommendations outlined in the protocols. It is speculated that they had modified their typical activities to some degree, which then felt like adherence to them. Presumably, their activity choices were guided by symptom relief and moderated by the conservative approach used in the CanChild protocols [3,4]. It also suggests that our arbitrary choice of 80% for a label of adherence was unrealistic, too high, and maybe even unnecessary. It was observed that participants who self-reported adherence to the RTA protocols achieved symptom resolution in a median of 13 days, and those who self-reported nonadherence achieved symptom resolution in 20 days. These data suggest that the mere presence of the protocols may alter behaviors, facilitating symptom resolution and reducing rates of reinjury as noted above.

The lack of adherence meant the youth were doing more than what the protocols recommended, which may seem contradictory to the low reinjury rate and symptom recovery patterns. However, existing evidence has shown that some cognitive and physical exertion early in recovery leads to shorter recovery times and syptom improvement [31,32,38-42]. In addition, Grool et al [43] examined 2413 youths with concussion and observed that physical activity within 7 days of acute injury was associated with reduced risk of persistent postconcussion symptoms [43]. Therefore, the nonadhering youth in our study were, in fact, getting some physical and cognitive activity early on. Yet the fact that they were not fully participating in activity may have contributed to the positive outcomes. The patterns demonstrated by the youth in this study provide valuable information for clinicians. They help define what activities and treatments are tolerable and acceptable for youth post concussion, meaning these are the levels of activity and treatment that youth can manage without exacerbating symptoms or causing further harm. Additionally, the study may indicate what is helpful, meaning the interventions or practices that contribute to positive outcomes and aid in the recovery process.

In light of these findings [8], along with data from our systematic review [44], the RTA/RTS protocols have been updated [5]. Some major revisions include a shortened rest period in stage 1 and the recommendation that youth progress through the stages before they are symptom free [5]. With these latest revisions, adherence of youth to the 2019 RTA/RTS protocols is expected to be greatly improved, although this requires further investigation.

This study is not without limitations. First, data on race and socioeconomic status were not collected. Second, adherence to RTA stages 1‐3, but not stages 4‐6, were assessed because only these stages had quantifiable physical activity cut points. Therefore, we were unable to objectively assess adherence to the later stages of the RTA protocol. Third, we accepted youth with concussion experiencing both acute and prolonged symptoms due to the nature of the research question. As such, the variability in time to symptom resolution and stage may be due to the prolonged symptoms of some participants. Finally, although we were able to retain the majority of participants, some were lost to follow-up or never achieved symptom resolution within the study period.

Overall, adherence to staged protocols post concussion was minimal according to the accelerometric data, but it was higher by self-report. More physical activity restrictions as specified in the RTA protocol, resulted in lower adherence. Although adherence was low, reinjury rate was lower than expected, suggesting a protective effect of being monitored and increased youth awareness of protocols. The results of this study support the move to less restrictive protocols and earlier resumption of daily activities that have since been implemented in more recent protocols.

## Abbreviations

BOT-2: Bruininks-Oseretsky Test of Motor Proficiency Second Edition
CDI: Children’s Depression Inventory
ImPACT: Immediate Post-Concussion and Cognitive Test
LPA: light physical activity
MRI: magnetic resonance imaging
MVPA: moderate-to-vigorous physical activity
PCSS: Post-Concussion Symptom Scale
REDCap: Research Electronic Data Capture
RTA: Return to Activity
RTS: Return to School

## References

[1] Langer L, Levy C, Bayley M (2020). Increasing incidence of concussion: true epidemic or better recognition?. J Head Trauma Rehabil.

[2] Ontario Agency for Health Protection and Promotion (Public Health Ontario) (2024). Injuries among Children Using the Canadian Health Survey on Children and Youth.

[3] DeMatteo C, Stazyk K, Giglia L (2015). A balanced protocol for return to school for children and youth following concussive injury. Clin Pediatr (Phila).

[4] DeMatteo C, Stazyk K, Singh SK (2015). Development of a conservative protocol to return children and youth to activity following concussive injury. Clin Pediatr (Phila).

[5] DeMatteo C, Randall S, Falla K (2020). Concussion management for children has changed: new pediatric protocols using the latest evidence. Clin Pediatr (Phila).

[6] McCrory P, Meeuwisse W, Dvořák J (2017). Consensus statement on concussion in sport-the 5th international conference on concussion in sport held in Berlin, October 2016. Br J Sports Med.

[7] McCrory P, Meeuwisse WH, Aubry M (2013). Consensus statement on concussion in sport: the 4th international conference on concussion in sport held in Zurich, November 2012. Br J Sports Med.

[8] DeMatteo CA, Lin CYA, Foster G (2021). Evaluating adherence to Return to School and Activity protocols in children after concussion. Clin J Sport Med.

[9] Gagnon I, Swaine B, Forget R (2009). Using activity diaries to measure children’s and adolescents’ compliance with activity restrictions after mild traumatic brain injury. J Head Trauma Rehabil.

[10] Jago R, Anderson CB, Baranowski T, Watson K (2005). Adolescent patterns of physical activity differences by gender, day, and time of day. Am J Prev Med.

[11] Moor HM, Eisenhauer RC, Killian KD (2015). The relationship between adherence behaviors and recovery time in adolescents after a sports-related concussion: an observational study. Int J Sports Phys Ther.

[12] Cain KL, Sallis JF, Conway TL, Van Dyck D, Calhoon L (2013). Using accelerometers in youth physical activity studies: a review of methods. J Phys Act Health.

[13] Corder K, Ekelund U, Steele RM, Wareham NJ, Brage S (2008). Assessment of physical activity in youth. J Appl Physiol (1985).

[14] Evenson KR, Catellier DJ, Gill K, Ondrak KS, McMurray RG (2008). Calibration of two objective measures of physical activity for children. J Sports Sci.

[15] Freedson P, Pober D, Janz KF (2005). Calibration of accelerometer output for children. Med Sci Sports Exerc.

[16] Reilly JJ, Penpraze V, Hislop J, Davies G, Grant S, Paton JY (2008). Objective measurement of physical activity and sedentary behaviour: review with new data. Arch Dis Child.

[17] Rowlands AV (2007). Accelerometer assessment of physical activity in children: an update. Pediatr Exerc Sci.

[18] Sirard JR, Pate RR (2001). Physical activity assessment in children and adolescents. Sports Med.

[19] Boshra R, Ruiter KI, DeMatteo C, Reilly JP, Connolly JF (2019). Neurophysiological correlates of concussion: deep learning for clinical assessment. Sci Rep.

[20] Ruiter KI, Boshra R, DeMatteo C, Noseworthy M, Connolly JF (2020). Neurophysiological markers of cognitive deficits and recovery in concussed adolescents. Brain Res.

[21] Dona O, Noseworthy MD, DeMatteo C, Connolly JF (2017). Fractal analysis of brain blood oxygenation level dependent (BOLD) signals from children with mild traumatic brain injury (mTBI). PLoS ONE.

[22] Ho RA, Hall GB, Noseworthy MD, DeMatteo C (2018). An emotional Go/No-Go fMRI study in adolescents with depressive symptoms following concussion. Int J Psychophysiol.

[23] Ho RA, Hall GB, Noseworthy MD, DeMatteo C (2020). Post-concussive depression: evaluating depressive symptoms following concussion in adolescents and its effects on executive function. Brain Inj.

[24] Berger I, Obeid J, Timmons BW, DeMatteo C (2017). Exploring accelerometer versus self-report sleep assessment in youth with concussion. Glob Pediatr Health.

[25] McCrory P, Meeuwisse W, Johnston K (2009). Consensus statement on concussion in sport: the 3rd international conference on concussion in sport held in Zurich, November 2008. J Athl Train.

[26] Harris PA, Taylor R, Thielke R, Payne J, Gonzalez N, Conde JG (2009). Research electronic data capture (REDCap)--a metadata-driven methodology and workflow process for providing translational research informatics support. J Biomed Inform.

[27] Lovell MR, Collins MW (1998). Neuropsychological assessment of the college football player. J Head Trauma Rehabil.

[28] Sady MD, Vaughan CG, Gioia GA (2014). Psychometric characteristics of the postconcussion symptom inventory in children and adolescents. Arch Clin Neuropsychol.

[29] Brown NJ, Mannix RC, O’Brien MJ, Gostine D, Collins MW, Meehan WP (2014). Effect of cognitive activity level on duration of post-concussion symptoms. Pediatrics.

[30] Fabre N, Lhuisset L, Bernal C, Bois J (2020). Effect of epoch length on intensity classification and on accuracy of measurement under controlled conditions on treadmill: towards a better understanding of accelerometer measurement. PLoS ONE.

[31] Kovacs M (2003). CDI 2. Children’s Depression Inventory 2. https://cad.storefront.mhs.com/collections/cdi-2.

[32] Ravens-Sieberer U, Gosch A, Rajmil L (2008). The KIDSCREEN-52 quality of life measure for children and adolescents: psychometric results from a cross-cultural survey in 13 European countries. Val Health.

[33] (2017). ImPACT. Baeline and post-injury testing for concussion care.

[34] Bruininks R, Bruininks B (2005). Bruininks-Oseretsky Test of Motor Proficiency.

[35] DeMatteo CA, Randall S, Lin CYA, Claridge EA (2019). What comes first: return to school or return to activity for youth after concussion? Maybe we don’t have to choose. Front Neurol.

[36] Ledoux AA, Barrowman NJ, Boutis K (2019). Multicentre, randomised clinical trial of paediatric concussion assessment of rest and exertion (PedCARE): a study to determine when to resume physical activities following concussion in children. Br J Sports Med.

[37] Huber DL, Thomas DG, Danduran M, Meier TB, McCrea MA, Nelson LD (2019). Quantifying activity levels after sport-related concussion using actigraph and mobile (mHealth) technologies. J Athl Train.

[38] Greco T, Ferguson L, Giza C, Prins ML (2019). Mechanisms underlying vulnerabilities after repeat mild traumatic brain injuries. Exp Neurol.

[39] Swaine BR, Tremblay C, Platt RW, Grimard G, Zhang X, Pless IB (2007). Previous head injury is a risk factor for subsequent head injury in children: a longitudinal cohort study. Pediatrics.

[40] Leddy JJ, Haider MN, Ellis M, Willer BS (2018). Exercise is medicine for concussion. Curr Sports Med Rep.

[41] Leddy JJ, Haider MN, Hinds AL, Darling S, Willer BS (2019). A preliminary study of the effect of early aerobic exercise treatment for sport-related concussion in males. Clin J Sport Med.

[42] Sawyer Q, Vesci B, McLeod TCV (2016). Physical activity and intermittent postconcussion symptoms after a period of symptom-limited physical and cognitive rest. J Athl Train.

[43] Grool AM, Aglipay M, Momoli F (2016). Association between early participation in physical activity following acute concussion and persistent postconcussive symptoms in children and adolescents. JAMA.

[44] DeMatteo C, Bednar ED, Randall S, Falla K (2020). Effectiveness of Return to Activity and Return to School protocols for children postconcussion: a systematic review. BMJ Open Sport Exerc Med.

